# Growth and Maturation Assessment in Youth Sport: Balancing Benefits and Risks

**DOI:** 10.1111/sms.70361

**Published:** 2026-08-17

**Authors:** Tommy R. Lundberg, Gemma N. Parry, Sean P. Cumming

**Affiliations:** ^1^ Division of Clinical Physiology, Department of Laboratory Medicine Karolinska Institutet Stockholm Sweden; ^2^ Unit of Clinical Physiology Karolinska University Hospital Stockholm Sweden; ^3^ Department for Health University of Bath Bath UK

**Keywords:** athlete development, body weight, injury risk, maturity, talent identification, team sports

## Abstract

The assessment and monitoring of growth and maturation (G&M) in youth sport has become increasingly debated, with some medical advisory boards expressing concerns that weighing young athletes may contribute to body image disturbances and eating disorders. In some instances, these concerns have prompted recommendations against anthropometric assessment in youth sport. This narrative review examines the evidence for and against G&M assessment in youth sport, evaluating the benefits of appropriately implemented assessment against the potential risks. G&M assessment supports injury prevention by identifying athletes undergoing rapid growth who are at increased risk of growth‐related and overuse injuries. It enables individualized training by aligning programing with biological rather than chronological age, and promotes inclusive talent identification by counteracting maturity‐related selection biases that systematically exclude late‐maturing athletes. While concerns about body image and eating disorders are valid, the reviewed evidence suggests that the risks are not associated with the collection of anthropometric measurements itself, but rather with how information is communicated and the broader sociocultural and coaching environments in which athletes train. Five G&M implementation principles are proposed to maximize benefits and minimize risks: stakeholder education, privacy protection, voluntary participation, assessment by trained professionals, and integration within a holistic and child‐centered athlete monitoring system. Based on the evidence reviewed, G&M assessment conducted within appropriate ethical and professional frameworks can support athlete welfare, development, and equitable participation.

## 1 Introduction

The assessment and monitoring of growth and maturation (G&M) in youth sport has become increasingly contested, particularly following recent statements, media reports, and professional and public commentary questioning the routine weighing of young athletes [1, 2]. Medical advisory boards in some countries, notably in Scandinavia, have raised concerns about the potential negative effects of weighing youth on body image and the development of body dysmorphia and/or eating disorders [3]. These concerns warrant serious consideration. A recent editorial has likewise called for thoughtful implementation of growth and maturity monitoring in high‐level youth football, drawing attention to both the potential rewards and the risks associated with frequent weighing of adolescents [4]. The present narrative review takes a broader view across youth sport contexts and synthesizes the evidence base supporting G&M assessment alongside its risks. We recognize at the outset that the concerns raised by clinicians, psychologists, athletes, and parents are legitimate and warrant serious consideration. Our aim, however, is to weigh these concerns against the potential benefits of assessment and monitoring, so that practice can be guided by a balanced appraisal of both.

Much of the opposition to weighing young athletes appears to stem partly from the International Olympic Committee's (IOC) consensus statement on Relative Energy Deficiency in Sport (REDs) [5], which in some instances has been interpreted as discouraging anthropometric assessment in youth sport more broadly. However, the consensus primarily addresses body composition monitoring in the context of identifying and managing REDs. Anthropometric measurements used for G&M assessment serve a different purpose: estimating biological maturation and monitoring normal growth trajectories, rather than evaluating body composition or weight status. When weight is measured as part of G&M assessment, it serves as an input to equations that estimate biological maturity status—not as an evaluation of body composition, weight status, or energy availability. Within these indirect equations, stature is the principal input, with body mass contributing to a lesser extent. We nonetheless give particular attention to weighing here because it, rather than the measurement of height, is the practice that has attracted controversy and is often perceived as more sensitive. Repeated measurements of weight also provide information on whether an athlete's growth trajectory is healthy and consistent with their maturation status, while deviations from expected growth or failure to show normal increases in body mass over time may serve as early indicators of growth‐related, autoimmune (i.e., coeliacs disease), or eating disorders. These distinctions have received limited attention in the current debate and warrant further consideration.

For the purposes of this narrative review, G&M assessment refers to the systematic measurement and monitoring of basic anthropometric characteristics, principally standing stature, sitting height, and body mass. These measurements are entered into validated equations to estimate biological maturity status, tempo, and timing. Commonly used indices include the maturity offset (an estimate of time from peak height velocity, PHV) [6] and the percentage of predicted adult height attained, the latter typically derived using the Khamis–Roche method [7]. These measurements are usually collected at regular intervals (e.g., every 3–6 months) during the circumpubertal years, and are used both in clinical growth monitoring and, increasingly, in youth sport to inform injury prevention, training individualization, and talent identification and development. Table 1 summarizes the common measures used, the indices they inform, the typical frequency of collection, and the contexts in which they are applied.

**TABLE 1:** Overview of measures and indices commonly used in growth and maturation (G&M) assessment, with typical methods, frequency of collection, and primary uses.

Measure/index	What it captures	Typical method	Typical frequency	Primary use
Standing stature	Linear growth status and velocity	Stadiometer	Every 3–6 months (every 6–8 weeks circa PHV)	Growth monitoring; input to maturity estimation
Sitting height	Trunk vs. leg growth status and velocity	Stadiometer and sitting height box	Every 3–6 months (every 6–8 weeks circa PHV) (every 6–8 weeks circa PHV)	Estimating maturity offset (proximity to PHV), maturity timing, growth rate of trunk and lower extremities
Body mass	Growth in mass; status and velocity	Calibrated scale, force plate (blinded option)	Every 3–6 months	Input to predicted adult height; growth monitoring (status and velocity)
Maturity offset	Estimated time before/after PHV	Predictive equation	Derived at each assessment	Identifying high‐risk growth windows and maturity timing; bio‐banding
Predicted adult height/% of predicted adult height	Biological maturity status relative to adult stature	Khamis–Roche method (child stature and body mass; mid‐parental height)	Derived at each assessment	Maturity status and timing; talent identification; bio‐banding
Skeletal age (where indicated)	Biological maturity (criterion method)	Radiographic assessment (specialist)	Infrequent	Reference/validation in selected cases

*Note:* Frequencies are indicative; exact protocols vary by sport and setting.

Abbreviation: PHV, peak height velocity.

At the same time, the potential benefits of G&M assessment extend beyond the clinical domain. Evidence from the fields of injury prevention, long‐term athlete development, and talent identification suggests that knowledge of maturation status, tempo and timing can inform training individualization, reduce injury risk during vulnerable growth periods, and promote more equitable selection and development practices. Routine anthropometric monitoring of this kind is already embedded in many elite settings: numerous professional football academies, including clubs in the English Premier League, and several national federations systematically record stature and body mass as part of player development and medical screening [8]. The Norwegian Football Federation, for example, has stated that its G&M screening program is conducted for medical purposes and serves goals of inclusion and injury prevention [9]. Whether such applications fall within or outside the scope of existing guidance remains a point of contention.

The purpose of this narrative review is to examine the evidence surrounding G&M assessment in youth sport, considering both the concerns that have been raised and the potential consequences of not assessing. In doing so, we review the roles of G&M assessment in injury prevention, training individualization, and inclusive talent identification, alongside the evidence on body image and eating disorder risk in athletic populations. We also examine the eating disorder prevention literature to evaluate whether anthropometric measurement for developmental purposes aligns with or contradicts established risk factors for disordered eating. Finally, we propose implementation principles to guide practice in ways that prioritize athlete welfare. To delimit the scope, this review concerns the use of anthropometric measurement for developmental (G&M) purposes; it engages the REDs and eating disorder literature only insofar as they contribute toward the discussion of the risk–benefit appraisal of assessment. The review also does not aim to provide a comprehensive account of REDs or of eating disorder etiology and treatment.

This article is a narrative review rather than a systematic review. A narrative approach was chosen because the relevant evidence spans several distinct domains, including injury epidemiology, growth and maturation, talent development, eating disorder prevention, and athlete welfare, that could not be captured adequately by a single systematic review question. Relevant literature was identified through a combination of database searching, citation tracking, and the authors' domain expertise, with priority given to longitudinal studies, reviews, consensus statements, and recent primary research. Because the review necessarily integrates heterogeneous and unevenly developed bodies of evidence, its limitations, including the potential for selection bias, are considered explicitly in a dedicated section.

## 2 Growth, Maturation, and Injury Risk

A central argument for G&M assessment in youth is injury prevention. The following section considers, in turn, the injury types that become more prevalent during periods of rapid growth and maturation, the strategies available to manage this risk, and how these considerations differ between male and female athletes. The association between growth, maturation, and injury risk in young athletes is well established [10]. During periods of rapid growth, particularly around the pubertal growth spurt and PHV, young athletes are more susceptible to certain types of injuries, especially growth‐related and overuse injuries. A scoping review of associations between growth, maturation, and injury in elite youth athletes found that injury incidence and burden generally increased with maturity status, although growth‐related injuries peaked during the adolescent growth spurt [10]. More rapid growth in stature and lower limb length was associated with higher injury incidence and burden, indicating biomechanical and musculoskeletal vulnerabilities that arise during rapid skeletal development.

Evidence from youth football demonstrates the practical relevance of these findings. The period during and 12 months after PHV represents a particularly vulnerable window for injury [11, 12, 13]. Research on youth football tournaments has found that athletes in the circa and post‐PHV stages who participated in congested schedules exhibited higher injury rates [14]. When practitioners identify which athletes are undergoing rapid growth, they can implement targeted symptom monitoring and prevention strategies to mitigate risk [15, 16]. These may include modified training loads and content appropriately scaled or implemented to the athlete's current physical development, specific strengthening exercises for at‐risk anatomical areas, and increased monitoring during high‐risk periods [16]. Practitioners may also encourage players to practice on cushioned and/or balance disruptive surfaces that limit the physical stress placed upon the body, challenge neuromuscular control and enhance lower‐limb stability [17]. In this context, maturation‐informed assessment operates as a developmental monitoring tool, helping practitioners align training load, recovery, and performance expectations with biological rather than chronological age. The potential to prevent even a small percentage of growth‐related injuries represents meaningful short‐ and long‐term health benefits that should be considered alongside the concerns associated with the measurement of weight.

The period of rapid growth requires particularly careful management. A prospective cohort study from a German elite youth football academy found that physeal injuries peaked in the U14 age group during the pubertal growth spurt, while the overall incidence and burden of injuries increased with age [18]. As the age at which individuals experience the pubertal growth spurt can vary by as much as 5 to 6 years [19], regular assessment of G&M is required to tailor training and competition loads accordingly. Thus, G&M assessment provides objective data that enables evidence‐informed decisions about training intensity, monitoring protocols, and preventive interventions. Without knowledge of individual maturation status, coaches and medical staff lack critical information regarding which athletes are approaching or currently within these high‐risk developmental windows. In this context, avoiding G&M assessment may not enhance safety, as it removes a key information source for injury prevention.

Knowing when an athlete is experiencing their growth spurt is also important for understanding their nutritional requirements. The demands of training combined with rapid growth may increase energy needs, and failure to meet these demands can lead to inadequate energy availability and, in severe cases, bone resorption [20]. This period also requires appropriate levels of protein and calcium, and particularly for girls, iron. G&M assessment thus serves not only injury prevention but also nutritional planning. A related consideration is that young athletes increasingly seek additional coaching outside their clubs, whether for speed training, technical skills, or other specialized areas. To avoid overloading athletes during vulnerable developmental periods, it is important that anyone working with the athlete has access to relevant maturation information. G&M assessment provides a basis for coordinating training loads across different training environments.

Finally, regarding sex differences, the onset of puberty represents a developmental stage at which ACL injury incidence rises more steeply in girls than boys [21]. This sex‐specific escalation is commonly linked to maturational changes in anatomy and neuromuscular function that accompany puberty, which may not be fully offset by current training or prevention practices [22].

## 3 Individualizing Development Through Maturation‐Informed Training

Beyond injury prevention, G&M assessment provides important data for individualizing training and development programs. As noted, young athletes of the same chronological age can differ substantially in their biological maturity status. The manner in which young athletes respond and adapt to different training stimuli varies according to maturation, reflecting maturity‐associated changes in the metabolic, endocrine, and neuromuscular systems. This variation is a key tenet of all long‐term athlete development models and why the regular assessment and monitoring of growth and maturation is recommended in these frameworks [23, 24].

One criticism raised in the public debate is that weight‐based measures are retrospective and cannot be used to prospectively predict the growth spurt. This characterization requires nuance. When integrated into methods such as the Khamis‐Roche equation [7], body mass is one of several inputs, alongside stature (the principal predictor) and mid‐parental height, used to predict adult stature. The percentage of predicted adult height attained then serves as an indicator of biological maturity status and, through serial measurement and comparison with reference data, can be used to characterize maturity timing and to approximate an athlete's proximity to PHV. Used in this way, repeated measurements allow practitioners to anticipate rather than merely react to periods of rapid growth [25, 26]. It is important to recognize, however, that these approaches yield estimates of maturational status with known error margins, and should be interpreted as guidance to inform clinical and coaching judgment rather than as precise individual assessments of developmental status [25]. When used appropriately, these tools are intended to guide practitioner awareness of development timing rather than to categorize or label athletes definitively. Thus, G&M assessment provides objective data that can inform individualized training and development. For early‐maturing athletes, this could mean focusing more deliberately on technical and tactical development rather than relying on physical dominance—an approach that may produce more complete, versatile athletes in the long term. For late‐maturing athletes, G&M assessment provides context and reassurance during a potentially challenging period and enables appropriately scaled expectations. Understanding that a late‐maturing athlete possesses genuine potential despite current physical disadvantages can transform how coaches interact with and develop that athlete. This individualization is important to athlete development. Rather than creating psychological burden, such an informed approach has the potential to foster motivation and reduce anxiety by helping young athletes understand their developmental trajectory and the reasons behind training modifications.

Research on strength and power development during the PHV period illustrates how maturation‐informed training can enhance outcomes. Sports training can mitigate performance decrements associated with adolescent awkwardness during the PHV period, and training characteristics such as frequency and sport specificity are identified as key moderating factors [27]. However, this benefit requires understanding each athlete's maturation status so that training can be appropriately calibrated. Athletes at different maturation stages may require different training emphases and workload management strategies. Programs that do not consider maturation status may risk either under‐challenging mature athletes or overwhelming immature athletes, potentially limiting developmental outcomes for both groups.

## 4 Inclusion and Equitable Opportunity

One of the most compelling arguments for G&M assessment is its role in promoting inclusion and equitable opportunity in youth sport. Research consistently demonstrates that early‐maturing athletes in youth sports often have temporary physical advantages compared to late‐maturing athletes [28], despite the latter potentially having equal or greater potential for long‐term athletic success [29, 30]. Selection and grouping based solely on chronological age can systematically disadvantage late‐maturing athletes, leading to de‐selection, dropout, or exclusion. For example, an analysis of over 1000 male soccer players in the Club Academy Scotland system found no late‐maturing players from age 14 onwards [31]. Such patterns raise concerns not only for talent development but also for public health, given that exclusion from organized sport may reduce opportunities for sustained physical activity engagement during developmentally sensitive years. By contrast, in some aesthetic or weight‐sensitive sports—such as competitive gymnastics, diving, and ballet—the selection bias may operate in the opposite direction, with later maturation sometimes favored [32]. These contrasting patterns further highlight the influence of maturation on opportunity structures across youth sport, and without G&M assessment there is a risk that children are excluded from participation in competitive sports based on attributes they have little control over. This is relevant to Article 28 of the United Nations Convention on the Rights of the Child, which states that all parties recognize the need for equitable access to educational activities that support healthy growth and learning, without discrimination [33].

Bio‐banding—the process of grouping athletes according to biological maturation rather than chronological age—has emerged as a promising complement to the traditional age group system providing more equitable athlete development and talent identification [34]. Multiple studies across diverse sports demonstrate the benefits of this approach. In youth football, handball, basketball and ice hockey, bio‐banding created more equitable groupings with homogenized physical attributes and performance characteristics [35, 36, 37, 38]. Players who were advanced in maturity for their age, yet physically matched when bio‐banded, experienced higher demands [37, 39], while late maturing players could utilize their skills to a greater extent—outcomes that support learning and development for all maturity groups. In addition, early maturing players faced higher physiological challenges in bio‐banded competition, while late‐maturing players gained substantially more opportunities to take on leadership roles and use and demonstrate their technical and tactical abilities.

The practical implications of assessing G&M extend beyond the grouping of athletes for competition. Several European countries have long incorporated biological maturity considerations into talent identification and development, and the Future Team concept—which creates separate national team pathways for late‐maturing athletes—has demonstrated success [40, 41]. Without objective G&M assessment, identifying these players for alternative development pathways would be considerably more difficult. Research has demonstrated that early maturation does not guarantee professional success; in fact, late matures appear relatively more likely to reach professional football when considering the overall conversion rate [30, 42]. This finding underscores the importance of continuing development opportunities for late‐maturing athletes and the role G&M assessment plays in enabling this inclusion.

Expert consensus supports this conclusion. A Delphi study with leading sport and exercise science practitioners in professional football academies achieved consensus that maturity‐related data collection is important for injury prevention, physical and performance‐related purposes, and for purposes beyond recruitment and retention decisions [43]. Panelists agreed that maturity‐related data collection is part of a multidisciplinary process dedicated to long‐term player development. This expert consensus suggests that the benefits of systematic maturation assessment are sufficiently established to warrant its continued application when implemented appropriately.

## 5 Addressing Legitimate Concerns

### 5.1 Prevalence and Context of Eating Disorder Risk in Athletes

The concerns about body image and eating disorders are legitimate and deserve careful attention. Research documents that body image concerns, disordered eating, and eating disorders occur among young athletes [44, 45]. Female athletes show particular vulnerability to eating disorder risk compared to males, and athletes competing in weight‐sensitive sports demonstrate elevated rates of eating pathology [46]. These findings are not trivial, and any intervention in sport must be implemented with genuine regard for mental health and wellbeing.

However, the distribution of eating disorder risk across sports is not uniform, and this context is important when evaluating concerns about G&M assessment. Reviews of the eating disorder literature indicate that the prevalence of disordered eating is highest in aesthetic sports, followed by weight‐class sports, while the prevalence in team sports is substantially lower [46, 47]. G&M assessment is, in our observation of current practice, currently most widely implemented in team sports such as football, handball, basketball, and ice hockey—the sport contexts where eating disorder prevalence is lowest. While this does not eliminate the need for caution, it does suggest that the concerns driving opposition to G&M assessment may be most relevant in sport contexts where such assessment is less commonly applied.

### 5.2 Established Risk Factors and the Role of Measurement

Understanding the drivers of eating disorder risk in athletes is essential for evaluating whether G&M assessment contributes to or is independent of that risk. The eating disorder prevention literature identifies two broad categories of risk factors: general factors that may place any individual at risk, and sport‐specific factors [47]. General predisposing factors include biological variables (age, genetics, pubertal status), psychological factors (body image dissatisfaction, low self‐esteem, perfectionism), and sociocultural factors (perceived pressure to conform to thinness ideals, family history of eating disorders). Sport‐specific factors include frequent weight regulation, external pressure to lose weight, lack of nutritional knowledge, the desire to be lean to improve performance, and the impact of coaching behavior [46, 47].

Prospective studies, which can establish temporal precedence and thus approach causal inference more closely than cross‐sectional designs, point consistently toward contextual and communicative factors rather than clinical measurement. A prospective controlled study of Norwegian athletes found that athletes who reported dieting and the desire to be leaner to improve performance were more likely to develop eating disorders [48]. A longitudinal investigation in aesthetic sports found that individual changes in the desire to be leaner to improve sports performance were predictive of disordered eating, and not vice versa [49]. Research on the coach–athlete relationship has identified conflict with coaches as a significant independent predictor of eating psychopathology, while coaching style characterized by body weight preoccupation has been found to increase dieting behavior, body image anxiety, and fear of fatness [50, 51]. Conversely, a supportive and caring coaching style may reduce eating disorder risk [50].

The eating disorder literature also describes a disordered eating continuum progressing from healthy dieting through extreme weight‐loss methods to clinical eating disorder, with established trigger factors including negative comments regarding body weight and shape [47]. Developmental G&M assessment—conducted by trained professionals, with results used as inputs to maturation‐estimation equations rather than as evaluations of body composition—does not directly engage the mechanisms identified along this continuum. The measurement itself appears not to be the risk; the risk lies in how weight‐related information is interpreted, communicated, and situated within the broader training environment. It should be acknowledged, however, that direct studies examining the psychological outcomes of developmental G&M assessment in youth sport are, to our knowledge, currently lacking. The present interpretation therefore rests largely on indirect evidence and on the absence of an identified causal mechanism linking developmental measurement to disordered eating, rather than on direct evidence of no effect.

### 5.3 The Distinction Between G&M Assessment and Body Composition Assessment

The distinction between G&M assessment and body composition assessment is important here (Table 2). Body composition assessment focuses on fat‐mass and related parameters—measurements that can foster a problematic focus on body fat reduction [52]. By contrast, G&M assessment uses anthropometric measurements as inputs to established equations for estimating maturation status. The purpose is developmental understanding, not body composition evaluation. When this distinction is clearly understood and communicated to athletes and parents, concerns about weight‐focused environments are substantially mitigated [53]. Research on eating disorders in athletes reveals that the primary risk factors involve sport‐specific pressures related to leanness ideals, coaching communication emphasizing weight or appearance, and individual psychological vulnerabilities—not the measurement of height and weight for developmental assessment and monitoring purposes [47]. Extending these recommendations to encompass developmental anthropometric assessment conflates two fundamentally different practices with different purposes, different methodologies, and, based on the available evidence, different risk profiles.

**TABLE 2:** Key distinctions between growth and maturation (G&M) assessment and body composition assessment.

Dimension	G&M assessment	Body composition assessment
Primary purpose	Estimate biological maturity status, tempo and timing; monitor healthy growth	Quantify fat mass and lean mass/relative adiposity
Measures used	Stature, sitting height, body mass, parental height	Skinfolds, bioimpedance, DXA, girths
Intended interpretation	Developmental stage relative to adulthood and to peers; proximity to PHV	Adiposity/leanness relative to reference or targets
Principal risks	Misclassification; labelling if poorly communicated	Weight/fat focus; leanness pressure; potential disordered‐eating triggers
Key safeguards	Trained assessors; private, non‐shaming protocols; education; voluntary participation	Strict clinical indication; clinical oversight; avoidance of leanness messaging
Intended data recipient	Medical/sport‐science staff; interpreted advice to coaches; athlete and parents	Clinician/practitioner under clinical governance

Abbreviations: DXA, dual‐energy X‐ray absorptiometry; PHV, peak height velocity.

### 5.4 Anthropometric Measurement as a Screening and Prevention Tool

An important observation from the eating disorder prevention literature is that anthropometric measurement, including height and weight, is itself recommended as part of preparticipation screening aimed at early identification of at‐risk athletes. Expert recommendations for eating disorder prevention in sport explicitly include height, weight, and related physical measurements in preparticipation examinations, alongside questions about nutrition, menstruation, and body image [47]. The rationale is that such measurements, when conducted by trained professionals, can help identify nutritional inadequacies, low energy availability, and early signs of disordered eating before they progress to clinical eating disorders. This represents a notable tension in the current debate: the same anthropometric measurements that some advisory boards seek to restrict in youth sport are recommended in the eating disorder prevention literature as tools for protecting athlete welfare.

Furthermore, routine clinician‐conducted weighing is generally recommended as part of the assessment and monitoring in evidence‐based treatments for eating disorders, particularly anorexia nervosa [54]. Weight monitoring is used to evaluate medical stability, track treatment responses, and set and review individualized weight restoration goals [55]. If clinical measurement of weight is considered appropriate and indeed necessary in the treatment of eating disorders, it follows that similar measurements conducted in a developmental context by trained professionals should not be categorically assumed to increase risk.

### 5.5 Supervised Versus Unsupervised Contexts

Young athletes are already exposed to body‐related discussions and comparisons that are a natural part of sport participation and adolescent development. The question is less about whether these issues will arise and more about how they are addressed. Ideally, they should be managed in structured, educational contexts rather than being shaped mainly by informal peer discussions, social media influences, or unverified sources of information [4]. Avoiding anthropometric assessment altogether may not prevent young people with eating disorders or body image concerns from weighing themselves. Instead, it risks shifting such behaviors into less supervised contexts, where professional guidance and safeguarding may be limited. Frequent self‐weighing behavior is common in individuals with eating disorders and is associated with greater eating disorder psychopathology [56]. This can lead to poor practices and removes the possibility of trained practitioners communicating information in a clinically informed, supportive and appropriate manner.

It is more appropriate to implement G&M screening programs that are supervised by trained medical practitioners who are equipped to communicate findings in an informed, supportive and constructive manner. Evidence indicates that well‐implemented, structured weighing programs, when combined with appropriate education and communication, can improve children's and families' understanding of normal growth and development processes [53, 57]. When young athletes understand that weight gain and physical changes are natural and necessary parts of healthy development, this knowledge can reduce rather than increase body‐related anxiety.

### 5.6 Education as a Protective Factor

Evidence from eating disorder prevention research further supports the role of education as a protective factor. A review of intervention studies testing prevention programs in female athletes found that educational initiatives consistently produced positive outcomes [47]. The ATHENA (Athletes Targeting Healthy Exercise and Nutrition Alternative) program, which delivered eight weekly sessions focused on healthy eating, exercise practices, and resistance to unhealthy weight‐control methods, found that intervention groups reported fewer health‐harming behaviors than control groups across multiple trials [58, 59]. Preventive educational work has been associated with a decreased prevalence of eating disorders [47]. Peer‐led interventions among female college athletes demonstrated reductions in dietary restraint, shape and weight concerns, and negative affect [60]. While the long‐term effectiveness of these programs requires further investigation, the available evidence suggests that structured education about nutrition, healthy development, and body image can reduce rather than increase eating disorder risk—supporting the position that informed, well‐communicated assessment is preferable to avoidance.

### 5.7 Interdisciplinary Dialogue

The differing perspectives in this debate highlight the need for interdisciplinary dialogue among advisory boards, researchers, and practitioners. Work relating to youth athlete welfare spans sports science, medicine, psychology, and education, with each domain contributing important, yet often discipline‐specific insights into the optimal support of developing athletes. When these perspectives operate largely in parallel rather than in collaboration, opportunities for a more holistic understanding may be missed. Moving forward, there is value not only in aligning established disciplines, but also in engaging with forms of knowledge and practice that are less commonly represented in this conversation, yet may offer equally meaningful contributions to how growth, development, and wellbeing are understood and supported in youth sport. Consensus positions formed primarily by researchers focused on singular subject areas or disciplines may not adequately consider other aspects of growth and maturation, including injury prevention and inclusive talent identification. A more balanced approach would integrate expertise across multiple disciplines.

It should also be acknowledged that researchers studying body image and eating disorders in sport are predominantly female, while the majority of those studying growth and maturation in sport have been male. This observation does not invalidate either perspective, but it underscores the importance of ensuring that recommendations are informed by diverse expertise, including clinicians with direct experience of working with injured developing athletes and those who understand both the risks and benefits of G&M assessment.

## 6 Implementing Best Practices

While the benefits of G&M assessment appear substantial, realizing these benefits while minimizing risks requires attention to implementation. G&M assessment should be guided by beneficence (it should be undertaken to benefit the athlete, through injury prevention, individualized development, and inclusion), non‐maleficence (it should be implemented so as to avoid foreseeable harm to body image and psychological wellbeing), autonomy (participation should be informed and voluntary), justice (it should promote equitable opportunity and challenge), and respect for privacy and child‐centered practice (the player's interests, dignity, and perspective should take precedence [4]). Table 3 summarizes these principles alongside practical examples and minimum safeguarding requirements and underscores that measurement alone is not the intervention; its benefits depend on appropriate interpretation, communication, and action. These ethical principles do not map one‐to‐one onto the five practical principles below; rather, each practical principle operationalizes one or more of them—voluntary participation (Principle 3) operationalizes autonomy, privacy and data governance (Principle 2) operationalizes non‐maleficence and privacy, stakeholder education and holistic integration (Principles 1 and 5) operationalize beneficence and justice, and assessment by trained professionals (Principle 4) operationalizes non‐maleficence and child‐centered practice. Based on the evidence reviewed, the following principles are proposed to guide implementation in ways that prioritize athlete welfare while preserving the developmental value of assessing and monitoring growth and maturation.

**TABLE 3:** Proposed implementation principles for G&M assessment, with practical examples and minimum safeguarding requirements.

Principle	Practical example	Minimum safeguard
1. Stakeholder education	Pre‐season workshop for athletes, parents and coaches on normal growth and development	All stakeholders receive education before assessment begins
2. Privacy and data governance	Blinded weighing: results shared individually and sensitively	No public or in‐team disclosure; defined data ownership, access, retention and handling on transfer/withdrawal
3. Voluntary participation	Clear, non‐coercive opt‐out offered at consent	Opt‐out available without penalty
4. Trained professionals	Assessment by qualified medical or sport‐science staff	Documented competence in both technical and psychological aspects
5. Integration	Maturity data combined with training load, performance evaluation, nutritional guidance, recovery and injury monitoring	Not used in isolation; embedded within a holistic, child‐centered monitoring system and multidisciplinary team

*Principle 1*. All stakeholders—athletes, parents, and coaches—should receive education about normal growth and development processes. This education should emphasize that growth‐related changes are positive indicators of healthy development, not problems to be managed or minimized. Such education represents a protective factor, increasing knowledge and awareness of growth and maturation whilst reducing rather than exacerbating anxiety about bodily changes [61].

*Principle 2*. Individual G&M data should never be made public or discussed in front of teammates. Measurements should be taken privately by qualified professionals and may incorporate structured non‐shaming protocols such as blinded weighing. Results should incorporate multidisciplinary input and be shared individually and sensitively with the athlete and parents. This protected approach preserves privacy and dignity while enabling the developmental and health benefits of assessment. Sound data governance is integral to this safeguard. Organizations should be explicit about who owns the data and who may access it, how long it is retained, and how it is handled when an athlete changes clubs or withdraws consent. As a general principle, coaches and non‐clinical staff should receive interpreted, actionable recommendations (e.g., that a player is in a high‐risk growth window) rather than raw values such as body mass, with raw data held by qualified medical or sport‐science personnel. We recognize that the operational specifics will appropriately be determined by individual clubs and organizations within the bounds of local data‐protection regulation.

*Principle 3*. Although G&M measurement is recommended for optimal development and safety, participation should remain genuinely voluntary. Athletes or parents who do not wish to participate should have clear, non‐coercive opt‐out options without penalty. This respect for autonomy is both ethically appropriate and practically wise, as it maintains trust in the sporting organization.

*Principle 4*. Assessment should only be carried out by trained professionals who understand both the technical aspects of maturity assessment and the psychological considerations of working with young athletes. The purpose and benefits should be clearly communicated, with emphasis on health, development, and performance optimization rather than body composition or weight control. Practitioners require adequate training and education to apply maturation assessment appropriately. Many academies recognize the importance of biological maturation yet fail to implement systematic assessment, suggesting that training and resource barriers persist [62]. Addressing these barriers through professional development is essential.

*Principle 5*. G&M assessment should be integrated into comprehensive athlete monitoring systems that track performance, training load, recovery, and injury patterns. In isolation, a single maturation assessment provides limited value. However, when integrated into a holistic developmental approach that considers multiple factors and perspectives, assessment becomes genuinely useful for individualizing programming and supporting long‐term athlete development.

There may also be a need to reframe the broader narrative around growth and maturation in young people rather than attempting to avoid the fact that children grow and develop. If a young athlete is thriving and developing, they should naturally be expected to gain weight, and this should be communicated and celebrated as a positive part of their normal development. Coaches need to understand this and communicate it constructively to both parents and athletes—a challenge many acknowledge and for which guidance would be welcomed. Research is currently underway to support practitioners and parents in having positive conversations around growth and development, and to gather athletes' perspectives on how they would like these discussions to occur. The goal should be to address growth and maturation as a natural and expected part of growing up, changing the conversation rather than avoiding it.

## 7 Limitations

Several limitations of this review should be acknowledged. First, as a narrative review it did not employ a systematic search protocol, and the selection of sources may therefore be subject to bias. We sought to mitigate this by drawing on multiple evidence domains and prioritizing consensus‐based and peer reviewed evidence, but the possibility of selective emphasis cannot be excluded. Second, the evidence base is uneven across domains: associations between maturation status and outcomes such as injury risk and selection bias are comparatively well established, whereas direct evidence that G&M assessment programs themselves improve injury, wellbeing, or developmental outcomes is more limited, and randomized intervention evidence is scarce. Our conclusions regarding the benefits of assessment should therefore be read as reasoned inferences from largely associational evidence. Third, and relatedly, direct studies of the psychological consequences of developmental G&M assessment in youth sport are currently lacking; the position that measurement itself is not the primary risk is consistent with the available evidence but cannot yet be regarded as established. Fourth, all methods used to estimate maturity status, including skeletal age, maturity offset equations and predicted adult height, carry known measurement error, particularly at the individual level, and misclassification could lead to inappropriate training modification, inaccurate reassurance, or unintended labelling. Growth and maturity assessment should be considered collectively as part of a broader measurement framework that informs, rather than determines, practice and should not be treated as deterministic categories. Finally, the evidence is heterogeneous across sports, age groups, and sexes, and important practical questions, including optimal measurement frequency, communication protocols, and the qualifications required of assessors, remain unresolved and warrant dedicated research.

## 8 Perspective

The assessment of growth and maturation in youth sport is a valuable tool for promoting athlete welfare when implemented appropriately. While legitimate concerns about potential negative effects on body image and eating disorder risk must be taken seriously, the evidence reviewed does not support categorical avoidance of G&M assessment as a risk‐free alternative. The established risk factors for eating disorders in athletes—including sport‐specific pressures related to leanness ideals, coaching communication emphasizing weight or appearance, and the desire to be leaner to improve performance—are contextually and communicatively driven and are not associated with clinical anthropometric measurement conducted for developmental purposes. Indeed, the eating disorder prevention literature itself recommends anthropometric measurement as part of preparticipation screening, and educational programs focused on healthy development have demonstrated protective effects against disordered eating.

The evidence reviewed supports the position that G&M assessment enables injury prevention during vulnerable developmental periods, facilitates individualized training and development, promotes inclusive talent identification and equitable opportunity, and supports long‐term athlete development across maturation trajectories. These benefits are substantial and well‐documented, and align broadly with recent calls for thoughtful, safeguarded implementation of G&M monitoring in elite youth football settings [4]. The risks, primarily related to body image concerns and eating disorder development, are real yet can be more effectively managed and mitigated through appropriate clinical intervention, education, and careful communication about the distinction between developmental assessment and body composition evaluation. Rather than defaulting to more reductive, albeit easily implemented, measures such as blanket bans on weighing, efforts to minimize body image disturbances and eating disorders in athletes should prioritize addressing the psychological and sociocultural determinants that drive these problems.

Young athletes deserve access to evidence‐based practices that can optimize their development, reduce their injury risk, and promote inclusive participation in sport. Based on the evidence reviewed, G&M assessment conducted by trained professionals, with proper safeguards and education, and with the explicit purpose of benefiting the child, can be consistent with athlete welfare when it is developmentally justified, voluntary, confidential, professionally delivered, and embedded within safeguarding and mental health–informed practice. The path forward involves ensuring that assessment is conducted with appropriate safeguards—comprehensive education programs, protection of privacy, voluntary participation options, and clear communication about purpose and benefits. The challenge is not whether to assess growth and maturation in youth sport, but how to do so in ways that genuinely serve athlete welfare and development.

## Funding

The authors have nothing to report.

## Conflicts of Interest

The authors declare no conflicts of interest.

## Data Availability

The authors have nothing to report.
